# Genetic and environmental contributions to the development of soft tissue facial profile: a twin study

**DOI:** 10.1093/ejo/cjae045

**Published:** 2024-09-13

**Authors:** Jamal Giri, Michelle Bockmann, Alan Brook, Angela Gurr, Toby Hughes

**Affiliations:** Faculty of Health and Medical Sciences, Adelaide Dental School, The University of Adelaide, Adelaide, SA 5000, Australia; Faculty of Health and Medical Sciences, Adelaide Dental School, The University of Adelaide, Adelaide, SA 5000, Australia; Faculty of Health and Medical Sciences, Adelaide Dental School, The University of Adelaide, Adelaide, SA 5000, Australia; Faculty of Health and Medical Sciences, Adelaide Dental School, The University of Adelaide, Adelaide, SA 5000, Australia; Faculty of Health and Medical Sciences, Adelaide Dental School, The University of Adelaide, Adelaide, SA 5000, Australia

**Keywords:** environment, facial profile, genetics, heritability, soft tissue, twins

## Abstract

**Objectives:**

This study aimed to determine the relative contribution of genetic and environmental factors in the phenotypic variation of the soft tissue facial profile during the mixed dentition and the permanent dentition stages.

**Methods:**

In this retrospective cohort study, standardized facial profile photographs of 139 twin pairs (55 monozygotic and 84 dizygotic) were obtained from archival records at the Adelaide Dental School. Photographic analysis used 12 angular and 14 linear facial profile measurements from the mixed dentition (7–11 years) to the permanent dentition (12–17 years) stages. A genetic analysis was performed using a univariate structural equation model adhering to the normal assumptions of a twin model.

**Results:**

In the mixed dentition stage, the additive genetic (A) and unique environment (E) model, AE model, was the most parsimonious in explaining the observed phenotypic variance for all 26 facial traits with the narrow-sense heritability estimates ranging between 0.38 and 0.79. In the permanent dentition, the AE model was the most parsimonious for 20 out of 26 traits, however, the variance of six traits, particularly those in the lower third of the face, was best explained by the shared environmental and unique environmental factors.

**Limitations:**

This study exclusively included twins of European ancestry.

**Conclusions:**

The soft tissue facial profile demonstrated dynamic genetic and environmental influences with a greater additive genetic influence during the mixed dentition and the early stages of the permanent dentition. However, there was evidence of increasing environmental influence in the lower third of the face during the early stages of the permanent dentition.

## Introduction

Facial morphology and attractiveness play a significant role in shaping both personal and professional experiences in life [1]. The desire to enhance facial aesthetics is one of the major motivators for orthodontic treatment [2]. This has led to a significant shift in orthodontics towards a soft tissue paradigm, prioritizing the attainment of a balanced facial soft tissue relationship over Angle’s paradigm which emphasized the skeletal and dental relationships [3].

The human face develops through a complex interactive process with multiple factors. This begins as early as week 4 post-conception and is not completed until adulthood, while minor changes continue to occur throughout life [4, 5]. The growth and development of the craniofacial structures are influenced by genetic and environmental factors, which interact over time [6, 7]. Thus, to predict or influence the development of a face, a better understanding of the factors underpinning facial development is crucial.

The study of human twins provides a foundation for investigating the genetic and environmental influences on dentofacial development [8]. The classical twin study design evaluates monozygotic (MZ) and dizygotic (DZ) twins raised in the same family environment [9]. Identical or MZ twins develop from a single fertilized ovum and are identical at the nucleotide level. Fraternal or DZ twins develop from two separately fertilized eggs and share on average 50% of the genes. The relative contribution of genetic and environmental factors on facial development can, therefore, be determined by comparing the phenotypic differences between the MZ and DZ twins.

The influence of genetic factors on craniofacial development was emphasized by Hunter [10], who concluded that vertical facial measurements were under greater genetic influence compared to horizontal measurements. Although several studies have since explored the genetic and environmental influence on craniofacial structures, the predominant focus of research has centered on the skeletal hard tissue, with limited attention given to the facial soft tissue [11–15]. One of the earliest studies analyzing the soft tissue profiles of twins found significant genetic contributions to facial convexity, facial height, and facial depth, while noting that variability in nose and lip morphology exhibited a greater environmental influence [16]. Conversely, a later study highlighted a strong genetic influence for the triangular area in the mid-face, including the nose [17]. This result was supported by a population-based twin study from the UK which revealed that genetic factors explained over 70% of the observed variation in facial size, nose characteristics, prominence of lips, and interocular distance [18]. The earlier findings were limited by the cross-sectional nature of the studies on the heritability of facial traits, as these traits change over time. In contrast, a recent longitudinal study found that soft tissue features of the face were influenced by either additive genetic, dominant genetic, or environmental factors [19]. The influence of dominant genetic factors was found to increase with age. However, this study focused solely on the soft tissue characteristics of the lower third of the face, providing a limited perspective on the overall facial characteristics [19]. In the absence of longitudinal twin studies evaluating the entire face, the relative contribution of genetic and environmental factors to the development of soft tissue characteristics of the face remains unclear.

Therefore, the aims of this study were to (1) partition the contribution of genetic and environmental factors in the phenotypic variation of the soft tissue facial profile and (2) assess the temporal change in the contribution of these factors during the development of the soft tissue facial profile from the mixed dentition stage to the permanent dentition stage.

## Materials and methods

### Study samples

**Figure 1.**
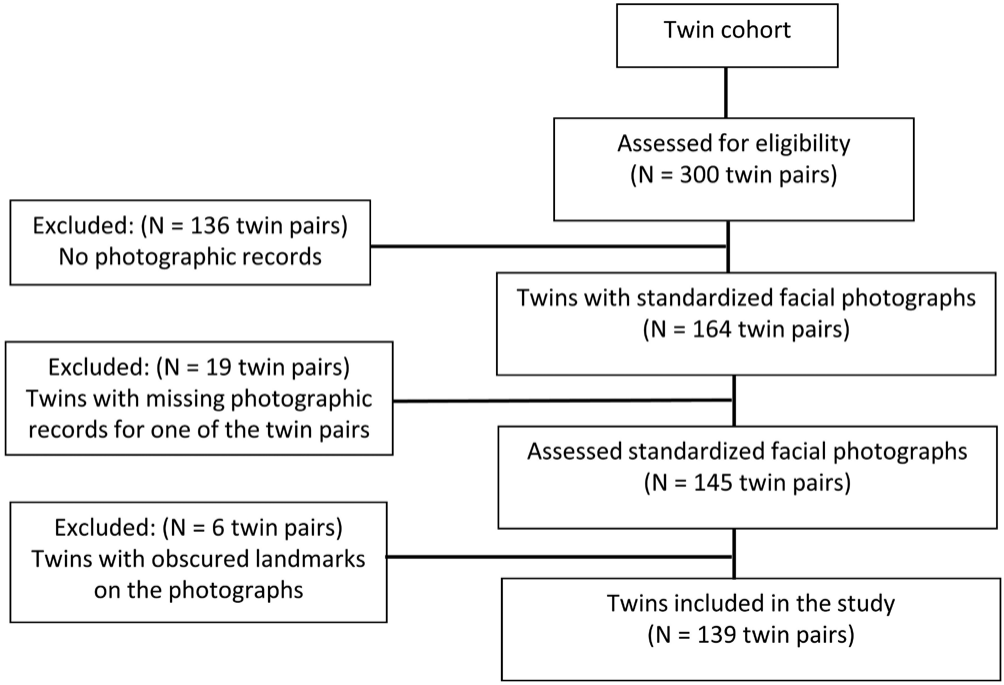
Flow diagram of participants’ eligibility criteria.

This retrospective cohort study obtained ethical approval from the Human Research Ethics Committee at the University of Adelaide (Approval number: H-2023-060). The population of interest was a national cohort of 300 twin pairs whose dental study models, facial photographs, and associated metadata were collected longitudinally between 1995 and 2006 by the Craniofacial Biology Research Group at the Adelaide Dental School [20]. A total of 394 facial profile photographs of 139 twin pairs (55 MZ—28 males and 27 females, 52 DZ—28 males and 24 females, and 32 DZ opposite-sex) between ages 7 and 17 years were obtained from the archives of the Craniofacial Biology Research Laboratory (Fig. 1). According to Visscher [21], a sample size of 96 twin pairs (48 MZ pairs and 48 DZ pairs) is required to reject the environmental hypothesis with a power of 0.95 when the heritability estimate is 0.6. Therefore, our sample size is deemed adequate, even though a sample size calculation was not performed due to constraints imposed by the size of the twin cohort. Of the 394 available photographs of the twins, 254 photographs (127 twin pairs) were taken during the mixed dentition stage, and 140 photographs (70 twin pairs) were taken during the permanent dentition stage. Photographs of 58 twin pairs were taken at both stages of dental development. All twins were of European ancestry, had not undergone previous orthodontic treatment, and did not exhibit craniofacial anomalies. The zygosities of the twins were confirmed by analyzing up to six highly variable genetic loci (FES, vWA31, F13A1, THO1, D21S11, and FGA) on six different chromosomes utilizing the DNA extracted from buccal cells. The probability of dizygosity, given concordance across all systems, was below 1%.

### Photographic analysis

**Figure 2.**
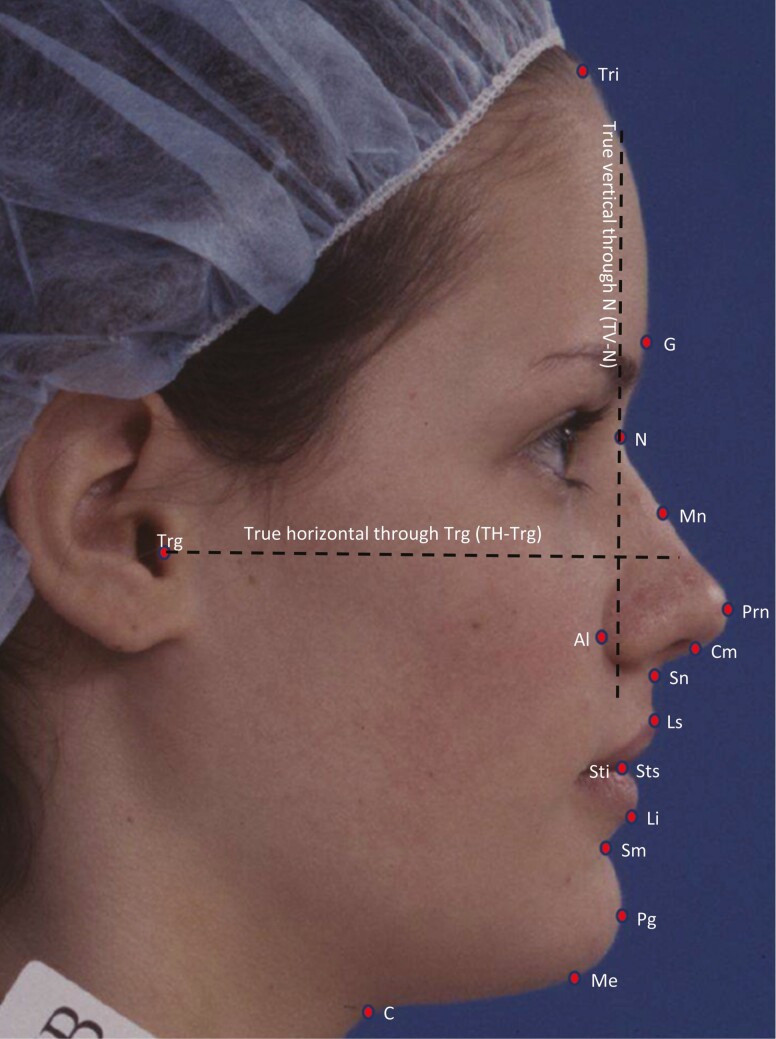
Facial landmarks and planes used in the study: trichion (Tri), glabella (G), nasion (N), mid nasal (Mn), pronasal (Prn), columella (Cm), subnasal (Sn), labial superior (Ls), stomium superior (Sts), stomium inferior (Sti), labial inferior (Li), supramental (Sm), pogonion (Pg), menton (Me), cervical (C), tragus (Trg), alare (Al), true vertical through nasion (TV-N), and true horizontal through tragus (TH-Trg).

**Figure 3.**
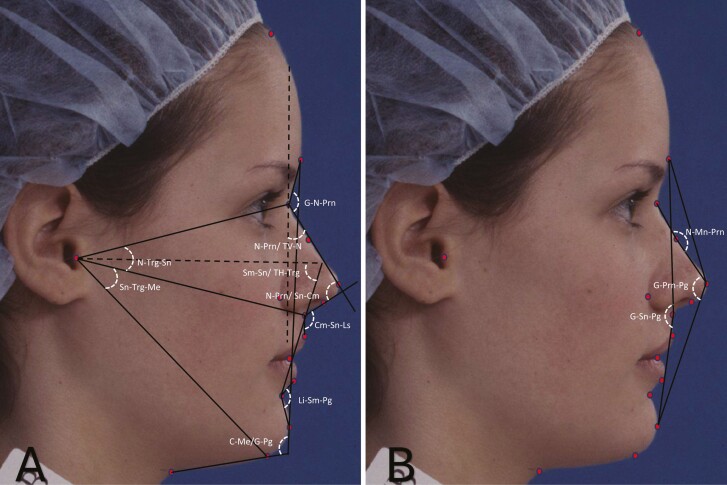
Angular facial measurements: (A) nasofrontal angle (G-N-Prn), vertical nasal angle (N-Prn/TV-N), nasolabial angle (Cm-Sn-Ls), mentolabial angle (Li-Sm-Pg), nasal angle (N-Prn/Sn-Cm), cervico-mental angle (C-Me/G-Pg), angle of middle facial third (N-Trg-Sn), angle of inferior facial third (Sn-Trg-Me), and angle of head position (Sm-Sn/TH-Trg), (B**)** angle of nasal dorsum (N-Mn-Prn), angle of facial convexity (G-Sn-Pg), and angle of total facial convexity (G-Prn-Pg).

**Figure 4.**
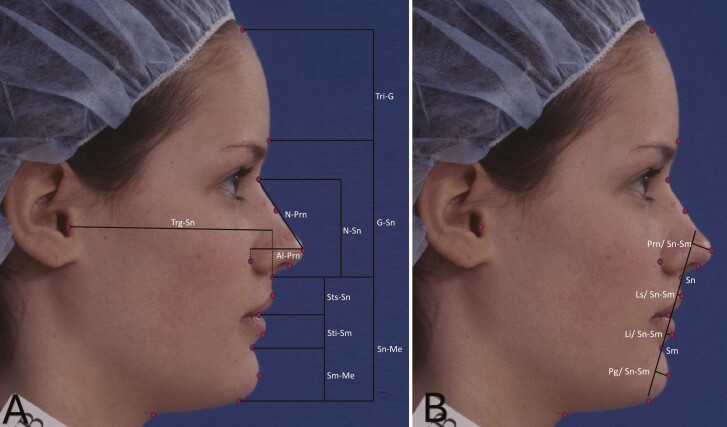
Linear facial measurements: (A**)** upper face length (Tri-G), mid face length (G-Sn), lower face length (Sn-Me), vertical nasal height (N-Sn), nasal bridge length (N-Prn), upper lip length (Sts-Sn), lower lip length (Sti-Sm), height of chin (Sm-Me), facial depth (Trg-Sn), and nasal depth (Al-Prn), (B**)** prominence of nose (Prn/ Sn-Sm), prominence of upper lip (Ls/Sn-Sm), prominence of lower lip (Li/Sn-Sm), and prominence of chin (Pg/Sn-Sm).

Profile photographs of the right side of the face of each individual were captured on a 35 mm ISO 200 Kodachrome slide film under a standardized condition in a natural head position [22]. The slide films were scanned at a high resolution (1200 DPI, 24-bit) on a flatbed scanner (EPSON Perfection V700) using a standard film holder to avoid image distortion. The scanned digital images were imported into the tpsDig2 software for landmark identification. Poor-quality photographs where the desired landmarks were obscured were excluded from the study. A total of 17 landmarks and two reference planes (true vertical and true horizontal) (Fig. 2) were identified on each photograph by a single investigator (JG) for analyzing facial characteristics. Twelve angular (Fig. 3) and 14 linear traits (Fig. 4) were measured using these landmarks, as per Fernandez-Riveiro *et al*. [23, 24], using a custom-written Python script.

### Method error

Intra- and inter-examiner reliability were calculated using the two-way mixed effects, absolute agreement, and single measurement model of intraclass correlation coefficient (ICC). Random error was estimated using the square root of the method of moments variance estimator (MME) [25]. Randomly selected photographs (50) were re-landmarked by the same investigator (JG) after 4 weeks to assess intra-operator reliability (ICC) and random error (MME). A second investigator (AG) also landmarked the same 50 photographs for re-calculation to assess the inter-operator reliability (ICC).

### Genetic analysis

**Figure 5.**
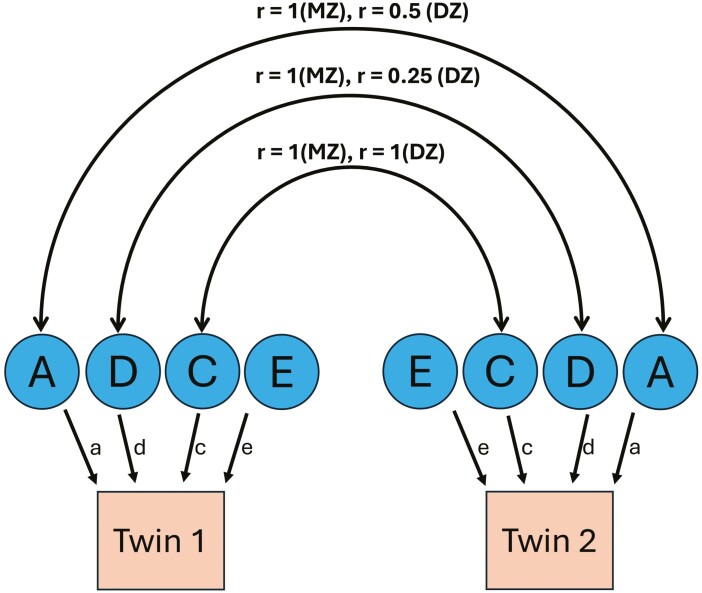
Univariate path diagram depicting potential influences on MZ and DZ twin pairs’ phenotypes: additive genetic factors (A), non-additive genetic factors (D), shared environmental factors (C), and unique environmental factors (E)-path coefficients (a, d, c, and e) indicate the relative significance of each influence, while double arrowheads signify correlation (r) between latent factors among twins.

ICCs were calculated between twin pairs for the angular and linear facial measurements. Genetic analysis was performed using a univariate structural equation model (SEM) with the OpenMx package in R [26]. The SEM operated under the assumption of random mating, equal trait-related shared environmental influences on both MZ and DZ twins, and the absence of gene-environment (GE) covariation or GE interaction [27]. Univariate ACE and ADE models were fitted to the angular and linear facial data of the twins to determine the contribution of additive genetic (A), non-additive genetic (D), shared environmental (C), and unique environmental (E) factors in the total phenotypic variance in the mixed and permanent dentition stages (Fig. 5). As C and D components are confounded in twins reared together, ACE and ADE models were fitted separately [28]. Akaike’s information criteria (AIC) and Chi-square likelihood ratio statistics were used to assess the model fit. The simplest model that could not be rejected based on the Chi-square likelihood value and with a low AIC was selected. Narrow-sense heritability estimates were reported for each trait, calculated as the ratio of additive genetic variation to the total phenotypic variation within the most parsimonious model. In addition, broad-sense heritability estimates were also calculated for the traits using Falconer’s formula [11].

Facial trait correlations were determined using the Pearson correlation coefficient, and differences in trait mean between sexes and twin zygosity were analyzed using Student’s *t*-test. Statistical analyses were performed in the R platform (4.2.0) and probabilities below 5% were considered statistically significant.

## Results

The ICC showed high intra-examiner (0.77–1.00) and inter-examiner (0.72–0.99) reliability for all measured traits. The random errors ranged from 0.5° to 2.6° for angular traits and from 0.4 mm to 1.1 mm for linear traits (Supplementary Document 1). The mean ages of the twins were 9.3 years (range: 7–11) for the mixed dentition and 14.4 years (range: 12–17) for the permanent dentition. With the exception of the mentolabial angle, lower face length, and facial depth among the 26 traits, no clinically significant differences, defined by values exceeding 2° or 2 mm [29], were observed between male and female twins (Supplementary Document 2). Consequently, pooled male and female twin data was used for subsequent analyses (Table 1). Except for one trait (angle of head position) in the mixed dentition and two traits (vertical nasal height and nasal bridge length) in the permanent dentition stages, there were no statistically significant differences between MZ and DZ twins. Facial traits displayed a range of correlations, with regional traits showing stronger correlations. Linear traits were more positively correlated with each other than angular traits (Supplementary Document 3).

**Table 1.** Mean values of the facial traits in the mixed and the permanent dentition stages.

Facial traits	Mixed dentition (Mean age = 9.3 years)		Permanent dentition (Mean age = 14.4 years)	
	n	Mean ± SD	n	Mean ± SD
Nasofrontal angle	127	139.8** ± **5.7°	70	141.5** ± **5.9°
Vertical nasal angle	127	28.9** ± **4.6°	70	31.9** ± **5.5°
Nasolabial angle	127	116.7** ± **9.7°	70	115.8** ± **10.1°
Mentolabial angle	127	145.1** ± **12.3°	70	139.5** ± **10.9°
Nasal angle	127	91.1** ± **8.0°	70	84.7** ± **9.0°
Angle of nasal dorsum	127	170.5** ± **4.7°	70	173.6** ± **4.2°
Cervico-mental angle	127	99.5** ± **7.2°	70	100.3** ± **7.3°
Angle of middle facial third	127	27.5** ± **2.1°	70	27.9** ± **1.6°
Angle of inferior facial third	127	38.2** ± **2.8°	70	36.6** ± **2.7°
Angle of head position	127	75.7** ± **5.4°	70	75.3** ± **5.3°
Angle of facial convexity	127	167.4** ± **4.3°	70	165.6** ± **4.9°
Angle of total facial convexity	127	145.5** ± **4.2°	70	139.9** ± **4.5°
Upper face length	127	36.5** ± **5.8 mm	70	42.0** ± **4.9 mm
Mid face length	127	56.6** ± **3.3 mm	70	62.9** ± **3.6 mm
Lower face length	127	55.2** ± **4.0 mm	70	61.2** ± **4.3 mm
Vertical nasal height	127	38.9** ± **2.6 mm	70	45.1** ± **3.0 mm
Nasal bridge length	127	32.6** ± **2.5 mm	70	39.1** ± **2.9 mm
Upper lip length	127	17.8** ± **1.8 mm	70	19.1** ± **1.9 mm
Lower lip length	127	14.0** ± **1.8 mm	70	16.1** ± **1.9 mm
Height of chin	127	22.8** ± **2.6 mm	70	25.8** ± **2.6 mm
Facial depth	127	82.1** ± **5.3 mm	70	94.9** ± **6.4 mm
Nasal depth	127	17.4** ± **2.3 mm	70	23.5** ± **2.6 mm
Prominence of nose	127	7.6** ± **1.4 mm	70	9.9** ± **1.9 mm
Prominence of upper lip	127	3.4** ± **1.1 mm	70	3.1** ± **1.1 mm
Prominence of lower lip	127	3.1** ± **1.3 mm	70	3.5** ± **1.3 mm
Prominence of chin	127	2.4** ± **1.3 mm	70	4.0** ± **1.9 mm

n: number of samples, SD: standard deviation.

The univariate genetic SEM in the mixed dentition stage found the additive genetic and unique environment (AE) model to be the most parsimonious in explaining the observed phenotypic variance for all 26 traits, with narrow-sense heritability estimates ranging between 0.38 and 0.79 (Table 2).

**Table 2.** Estimates of variance components and 95% confidence intervals for facial traits in the mixed dentition.

Facial traits	rMZ	rDZ	Model	A	95% CI	E	95% CI	h^2^	H^2^
Nasofrontal angle	0.79	0.27	AE	0.77	0.63–0.85	0.23	0.14–0.36	0.77	1.04
Vertical nasal angle	0.48	0.27	AE	0.52	0.31–0.67	0.48	0.32–0.68	0.52	0.42
Nasolabial angle	0.64	0.21	AE	0.62	0.48–0.72	0.38	0.28–0.52	0.62	0.86
Mentolabial angle	0.46	0.31	AE	0.50	0.29–0.64	0.50	0.35–0.70	0.50	0.30
Nasal angle	0.70	0.27	AE	0.69	0.58–0.80	0.31	0.20–0.41	0.69	0.86
Angle of nasal dorsum	0.62	0.16	AE	0.60	0.40–0.74	0.40	0.25–0.59	0.60	0.92
Cervico-mental angle	0.73	0.52	AE	0.73	0.61–0.81	0.27	0.18–0.38	0.73	0.42
Angle of middle facial third	0.48	0.24	AE	0.49	0.26–0.65	0.51	0.34–0.73	0.49	0.48
Angle of inferior facial third	0.28	0.25	AE	0.38	0.14–0.56	0.62	0.43–0.85	0.38	0.06
Angle of head position	0.70	0.25	AE	0.70	0.54–0.80	0.30	0.19–0.45	0.70	0.90
Angle of facial convexity	0.67	0.22	AE	0.64	0.46–0.76	0.36	0.23–0.53	0.64	0.90
Angle of total facial convexity	0.76	0.32	AE	0.71	0.57–0.80	0.29	0.19–0.42	0.71	0.88
Upper face length	0.56	0.23	AE	0.51	0.32–0.64	0.49	0.35–0.67	0.51	0.66
Mid face length	0.72	0.38	AE	0.75	0.62–0.83	0.25	0.16–0.37	0.75	0.68
Lower face length	0.64	0.44	AE	0.69	0.54–0.79	0.31	0.21–0.45	0.69	0.40
Vertical nasal height	0.72	0.31	AE	0.71	0.55–0.81	0.29	0.18–0.44	0.71	0.82
Nasal bridge length	0.77	0.49	AE	0.79	0.67–0.85	0.21	0.14–0.32	0.79	0.56
Upper lip length	0.53	0.24	AE	0.55	0.34–0.70	0.45	0.30–0.65	0.55	0.58
Lower lip length	0.58	0.35	AE	0.64	0.45–0.75	0.36	0.24–0.54	0.64	0.46
Height of chin	0.58	0.19	AE	0.57	0.35–0.71	0.43	0.28–0.64	0.57	0.78
Facial depth	0.47	0.16	AE	0.41	0.17–0.59	0.59	0.41–0.82	0.41	0.62
Nasal depth	0.56	0.24	AE	0.49	0.29–0.63	0.51	0.36–0.70	0.49	0.64
Prominence of nose	0.59	0.33	AE	0.62	0.44–0.74	0.38	0.25–0.55	0.62	0.52
Prominence of upper lip	0.51	0.25	AE	0.54	0.33–0.69	0.46	0.30–0.66	0.54	0.52
Prominence of lower lip	0.45	0.29	AE	0.51	0.29–0.67	0.49	0.32–0.70	0.51	0.32
Prominence of chin	0.60	0.40	AE	0.64	0.48–0.75	0.36	0.24–0.51	0.64	0.40

ICC for MZ twins (rMZ), ICC for DZ twins (rDZ), additive genetic variance (A), unique environmental variance (E), confidence interval (CI), narrow-sense heritability (h^2^), and broad-sense heritability (H^2^).

In the permanent dentition stage, the AE model was the most parsimonious for 20 out of 26 traits with narrow-sense heritability estimates ranging between 0.39 and 0.83. However, the shared environment and unique environment (CE) model was found to be the most parsimonious for the remaining six traits, namely, mentolabial angle, angle of inferior facial third, upper face length, lower face length, prominence of lower lip, and nasal bridge length (Table 3).

**Table 3.** Estimates of variance components and 95% confidence intervals for facial traits in the permanent dentition.

Facial traits	rMZ	rDZ	Model	A	95% CI	C	95% CI	E	95% CI	h^2^	H^2^
Nasofrontal angle	0.73	0.47	AE	0.75	0.57–0.85	–	–	0.25	0.14–0.42	0.75	0.52
Vertical nasal angle	0.32	0.26	AE	0.39	0.06–0.63	–	–	0.61	0.36–0.93	0.39	0.12
Nasolabial angle	0.64	0.21	AE	0.60	0.34–0.73	–	-	0.40	0.26–0.65	0.60	0.86
Mentolabial angle	0.55	0.42	CE	–	–	0.44	0.23–0.59	0.56	0.40–0.76	–	0.26
Nasal angle	0.61	0.33	AE	0.64	0.39–0.75	–	–	0.36	0.24–0.60	0.64	0.56
Angle of nasal dorsum	0.51	0.27	AE	0.51	0.23–0.70	–	–	0.49	0.29–0.76	0.51	0.48
Cervico-mental angle	0.44	0.27	AE	0.61	0.27–0.79	–	–	0.39	0.20–0.72	0.61	0.34
Angle of middle facial third	0.70	0.15	AE	0.59	0.29–0.76	–	–	0.41	0.23–0.70	0.59	1.1
Angle of inferior facial third	0.55	0.50	CE	^–^	–	0.51	0.32–0.66	0.49	0.33–0.67	^–^	0.1
Angle of head position	0.45	0.23	AE	0.41	0.15–0.62	–	–	0.59	0.37–0.85	0.41	0.44
Angle of facial convexity	0.68	0.30	AE	0.68	0.44–0.81	–	–	0.32	0.18–0.55	0.68	0.76
Angle of total facial convexity	0.81	0.33	AE	0.80	0.64–0.86	–	–	0.20	0.13–0.35	0.80	0.96
Upper face length	0.45	0.46	CE	–	–	0.46	0.25–0.60	0.54	0.39–0.74	–	−0.02
Mid face length	0.59	0.33	AE	0.61	0.35–0.76	–	–	0.39	0.23–0.64	0.61	0.52
Lower face length	0.54	0.59	CE	–	–	0.57	0.39–0.71	0.43	0.28–0.60		−0.10
Vertical nasal height	0.70	0.23	AE	0.67	0.43–0.80	–	–	0.33	0.19–0.56	0.67	0.94
Nasal bridge length	0.70	0.53	CE	–	–	0.62	0.45–0.74	0.38	0.25–0.54	–	0.34
Upper lip length	0.48	0.35	AE	0.52	0.23–0.70	–	–	0.48	0.29–0.76	0.52	0.26
Lower lip length	0.46	0.35	AE	0.57	0.26–0.75	–	–	0.43	0.24–0.73	0.57	0.22
Height of chin	0.60	0.31	AE	0.57	0.33–0.73	–	–	0.43	0.26–0.66	0.57	0.58
Facial depth	0.73	0.48	AE	0.77	0.59–0.84	–	–	0.23	0.15–0.40	0.77	0.50
Nasal depth	0.66	0.44	AE	0.71	0.52–0.83	–	–	0.29	0.17–0.47	0.71	0.44
Prominence of nose	0.55	0.19	AE	0.50	0.23–0.69	–	–	0.50	0.30–0.76	0.50	0.72
Prominence of upper lip	0.56	0.30	AE	0.63	0.36–0.79	–	–	0.37	0.20–0.63	0.63	0.52
Prominence of lower lip	0.21	0.28	CE	–	–	0.24	0.01–0.44	0.76	0.55–0.99	–	−0.14
Prominence of chin	0.87	0.22	AE	0.83	0.69–0.90	–	–	0.17	0.09–0.30	0.83	1.3

ICC for MZ twins (rMZ), ICC for DZ twins (rDZ), additive genetic variance (A), shared environmental variance (C), unique environmental variance (E), confidence interval (CI), narrow-sense heritability (h^2^), and broad-sense heritability (H^2^).

## Discussion

In this study, 12 angular and 14 linear facial traits were analyzed in 139 twin pairs from the mixed to the permanent dentition stages to determine the contribution of genetic and environmental factors in the phenotypic variation of the soft tissue facial profile using SEM. The AE model was the most parsimonious for explaining the soft tissue facial profile variance in both stages of dentition except for six traits (angle of inferior facial third, mentolabial angle, lower face length, prominence of lower lip, upper face length, and nasal bridge length) in the permanent dentition stage.

For most of the traits in the mixed and the permanent dentition stages, ICC between MZ twin pairs was approximately twice those of DZ twin pairs, suggesting an additive genetic influence. MZ twin pair correlations exceeded twice the DZ twin pair correlations for some traits, indicating genetic dominance. The power of the sample was sufficient to detect additive genetic influence but might not be sufficient to detect genetic dominance for twins raised together [30]. However, for the aforementioned six traits, the MZ and DZ correlations were nearly equal, suggesting a greater environmental influence. While we have also reported broad-sense heritability estimates for potential comparison with studies that solely report such estimates, caution is advised in their interpretation due to the likelihood of spurious values.

Facial traits with high correlations did not always have similar heritability estimates. For instance, in the permanent dentition stage, the facial depth, and nasal depth displayed a positive correlation and had similar narrow-sense heritability estimates of 0.77 and 0.71, respectively. However, the lower face length, which the CE model best explained, and the height of chin (AE model) also exhibited a strong positive correlation. This suggests that the phenotypic correlation among facial traits cannot serve as a proxy for genetic correlation.

Unlike Harris and Johnson [31], who found an increase in heritability estimates for craniofacial skeletal traits from age 4 to 14 years, which plateaued thereafter, not all soft tissue traits in the facial profile exhibited the same trend. In our study, the narrow-sense heritability estimates for eight traits related to the mid-face, nose, and chin were all above 0.6 at both time points, indicating a strong genetic influence on these facial regions. Earlier studies had suggested a strong genetic influence on the nose [18, 32]. While our results broadly supported the earlier findings for most nose-related traits, we also observed an increasing environmental influence on the nasal bridge length in the permanent dentition stage. Another significant finding was the notable rise in heritability estimates for facial depth and nasal depth during the permanent dentition stage, suggesting an increasing genetic influence on the depth of the face with age.

Few studies have evaluated the genetic and environmental influences on craniofacial structures using SEM. Our finding that the AE model provided the most parsimonious explanation of variance in facial soft tissue aligns with earlier studies which also identified the AE model as the best-fitting model for explaining the variance in skeletal craniofacial morphology [14, 33]. As is the finding that the shared environmental and unique environmental influences explain the variance for some skeletal traits. Carels *et al*. [14], reported a strong genetic influence on mandibular body length in the chin region, with the heritability estimate above 0.8. Although not directly comparable due to variable disparities, our study’s finding that soft tissue chin prominence exhibited heritability estimates ranging from 0.64 to 0.83 across the mixed and the permanent dentitions, indicates a significant genetic role in chin development. Furthermore, the heritability estimates of the soft tissues reported in our study are comparable to those for dental arch lengths and widths [34]. It is possible, therefore, that both hard and soft tissues of the craniofacial region are influenced by similar genetic and environmental factors.

The findings of our study can be meaningfully compared with those of Hersberger-Zurfluh *et al*. [19], who also evaluated the soft tissue profile of twins using SEM. Unfortunately, that study only assessed seven traits which may not sufficiently explain the complexity of the facial profile. Hersberger-Zurfluh *et al*. [19], also found a significant genetic influence on the facial profile with facial convexity and nose prominence best explained by the AE model. However, unlike our study, they identified the ADE model as the most parsimonious for the remaining five traits with increasing dominant genetic influence from ages 12 to 17 [19]. An important finding of our study was a shift in the best-fitting model from AE to CE in the permanent dentition stage for six traits (angle of inferior facial third, mentolabial angle, lower face length, prominence of lower lip, upper face length, and nasal bridge length) indicating an increased environmental influence. An opportunity was missed as we could not compare the genetic and environmental influences on these six traits in the existing literature, as they had not been evaluated previously. Although earlier studies have evaluated the growth changes in facial soft tissue among twins, they assessed only a limited number of facial traits [19, 35]. The present study adds estimates of the additive genetic, shared environmental, and unique environmental components for many facial traits. Future research on facial soft tissue should assess sufficient standard traits to explore its complexity and use appropriate genetic analysis to enable cross-study comparisons.

Four out of six traits, best explained by the CE model, described the lower facial third, suggesting a growing environmental influence on this region of the face with age. This could be a promising finding because orthodontic therapy usually influences the structures on the lower facial third [36]. As orthodontic intervention itself is considered an environmental factor [37], timing the orthodontic intervention during periods of greater environmental influence on soft tissue could enhance the treatment outcome. This strengthens the case for single-phase orthodontic treatment during permanent dentition over the two-phase approach [38]. The findings suggest that delaying orthodontic intervention until the early stage of the permanent dentition could yield a more favorable soft tissue response than initiating treatment during the mixed dentition stage.

During the mixed dentition and the early stages of the permanent dentition, children usually undergo orthodontic screening and may receive the most orthodontic/orthopedic interventions [39]. As facial traits with a greater environmental influence are typically expected to exhibit a more favorable response to orthodontic intervention [13], a better understanding of genetic and environmental influences on the facial soft tissue during this period helps plan an appropriate timing for intervention, set realistic soft tissue expectations, and acknowledge the limitations of orthodontic therapy. The findings of this study will assist orthodontists in predicting soft tissue facial growth, allowing them to customize treatment timing for optimal results. This study also lays the groundwork for genome-wide association studies on several facial traits with substantial genetic components.

There are some limitations to this study. This study exclusively included twins of European ancestry, potentially restricting the generalizability of its findings to broader populations. In the absence of lateral cephalograms, the facial soft tissue growth of the twins could not be correlated with their stages of skeletal maturation. Furthermore, it was a two-dimensional (2D) photographic assessment of a three-dimensional (3D) structure. Despite the advent of 3D imaging, 2D facial photographs are still routinely used in orthodontic treatment and clinicians express greater confidence in using 2D images for diagnosis [40]. However, further research evaluating 3D facial images on a longitudinal twin sample is necessary to enhance our understanding of the genetic, environmental, and epigenetic influences on the soft tissue facial profile.

## Conclusions

The soft tissue facial profile demonstrated dynamic genetic and environmental influences from the mixed dentition to the permanent dentition stage, spanning ages 7 to 17 years. During the mixed dentition and the early stages of permanent dentition, the soft tissue facial profile was significantly influenced by both additive genetic and unique environmental factors. Narrow-sense heritability estimates for eight traits related to the mid-face, nose, and chin were above 0.6 at both time points, suggesting a strong genetic influence on these facial regions. However, there was evidence of increasing shared environmental and unique environmental influences on some traits, particularly on the lower third of the face (such as angle of inferior facial third, mentolabial angle, lower face length, and prominence of lower lip) during the early stages of the permanent dentition. The subsequent phase of research will integrate multivariate genetic analysis with geometric morphometric facial analysis to elucidate the genetic and environmental interplay during soft tissue facial profile development.

## Supplementary Material

cjae045_suppl_Supplementary_Material

## Data Availability

The data will be made available upon reasonable request to the corresponding author.
