# Supplementary material for: Genetic and environmental contributions to the development of soft tissue facial profile: a twin study
Source: Eur J Orthod. 2024 Sep 13;46(5):cjae045. doi: 10.1093/ejo/cjae045 (PMC11398901; doi:10.1093/ejo/cjae045)

**Supplementary document 1: Systematic and random errors for the measured facial traits**

| **Facial traits** | **Intra-examiner ICC** | **Inter-examiner ICC** | **Random error (MME)** |
| --- | --- | --- | --- |
| Nasofrontal angle | 0.91 | 0.86 | 1.81° |
| Vertical nasal angle | 0.98 | 0.92 | 0.70° |
| Nasolabial angle | 0.79 | 0.88 | 2.66° |
| Mentolabial angle | 0.89 | 0.79 | 2.54° |
| Nasal angle | 0.86 | 0.88 | 2.61° |
| Angle of nasal dorsum | 0.81 | 0.73 | 2.33° |
| Cervico-mental angle | 0.97 | 0.94 | 1.04° |
| Angle of middle facial third | 0.91 | 0.80 | 0.52° |
| Angle of inferior facial third | 0.90 | 0.72 | 0.80° |
| Angle of head position | 0.99 | 0.98 | 0.61° |
| Angle of facial convexity | 0.94 | 0.93 | 0.83° |
| Angle of total facial convexity | 0.98 | 0.97 | 0.57° |
| Upper face length | 0.92 | 0.77 | 1.18 mm |
| Mid face length | 0.96 | 0.94 | 0.78 mm |
| Lower face length | 0.97 | 0.96 | 0.77 mm |
| Vertical nasal height | 0.96 | 0.86 | 0.80 mm |
| Nasal bridge length | 0.97 | 0.91 | 0.67 mm |
| Upper lip length | 0.93 | 0.88 | 0.57 mm |
| Lower lip length | 0.95 | 0.83 | 0.43 mm |
| Height of chin | 0.93 | 0.89 | 0.73 mm |
| Facial depth | 1.00 | 0.99 | 0.41 mm |
| Nasal depth | 0.97 | 0.97 | 0.54 mm |
| Prominence of nose | 0.93 | 0.93 | 0.50 mm |
| Prominence of upper lip | 0.77 | 0.81 | 0.45 mm |
| Prominence of lower lip | 0.86 | 0.84 | 0.37 mm |
| Prominence of chin | 0.94 | 0.94 | 0.39 mm |

ICC: Intraclass correlation coefficient, MME: Square root of the method of moments variance estimator

**Supplementary document 2: Comparison of mean values of facial traits between male and female twins**

| **Facial traits** | **Sex** | **n** | **Mean** | **SD** | **Mean difference** | **t** | **p-value** |
| --- | --- | --- | --- | --- | --- | --- | --- |
| Nasofrontal angle  (°) | male | 100 | 140.54 | 5.67 | 0.18 | 0.21 | 0.83 |
|  | female | 97 | 140.36 | 6.09 |  |  |  |
| Vertical nasal angle  (°) | male | 100 | 30.79 | 5.10 | 1.62 | 2.22 | 0.03 |
|  | female | 97 | 29.17 | 5.19 |  |  |  |
| Nasolabial angle  (°) | male | 100 | 117.25 | 9.45 | 1.64 | 1.17 | 0.24 |
|  | female | 97 | 115.62 | 10.23 |  |  |  |
| Mentolabial angle  (°) | male | 100 | 141.99 | 12.38 | -2.32^#^ | -1.35 | 0.18 |
|  | female | 97 | 144.31 | 11.83 |  |  |  |
| Nasal angle  (°) | male | 100 | 89.92 | 9.45 | 2.00 | 1.62 | 0.11 |
|  | female | 97 | 87.88 | 8.24 |  |  |  |
| Angle of nasal dorsum  (°) | male | 100 | 172.14 | 4.48 | 1.02 | 1.50 | 0.14 |
|  | female | 97 | 171.12 | 5.05 |  |  |  |
| Cervico-mental angle  (°) | male | 100 | 99.92 | 6.91 | 0.21 | 0.20 | 0.84 |
|  | female | 97 | 99.71 | 7.72 |  |  |  |
| Angle of middle facial third  (°) | male | 100 | 27.76 | 1.69 | 0.11 | 0.40 | 0.69 |
|  | female | 97 | 27.65 | 2.26 |  |  |  |
| Angle of inferior facial third  (°) | male | 100 | 38.11 | 2.64 | 0.92 | 2.23 | 0.03 |
|  | female | 97 | 37.19 | 3.15 |  |  |  |
| Angle of head position  (°) | male | 100 | 75.85 | 5.29 | 0.48 | 0.62 | 0.53 |
|  | female | 97 | 75.37 | 5.54 |  |  |  |
| Angle of facial convexity  (°) | male | 100 | 165.98 | 3.94 | -1.64 | -2.52 | 0.01 |
|  | female | 97 | 167.62 | 5.13 |  |  |  |
| Angle of total facial convexity  (°) | male | 100 | 142.87 | 5.01 | -1.39 | -1.90 | 0.06 |
|  | female | 97 | 144.26 | 5.22 |  |  |  |
| Upper face length  (mm) | male | 100 | 38.06 | 6.65 | -0.86 | -0.98 | 0.33 |
|  | female | 97 | 38.92 | 5.53 |  |  |  |
| Mid face length  (mm) | male | 100 | 59.23 | 4.69 | 0.71 | 1.09 | 0.28 |
|  | female | 97 | 58.52 | 4.46 |  |  |  |
| Lower face length  (mm) | male | 100 | 58.87 | 5.11 | 3.00^#^ | 4.37 | <0.01 |
|  | female | 97 | 55.87 | 4.48 |  |  |  |
| Vertical nasal height  (mm) | male | 100 | 41.71 | 4.17 | 1.20 | 2.08 | 0.04 |
|  | female | 97 | 40.51 | 3.91 |  |  |  |
| Nasal bridge length  (mm) | male | 100 | 35.60 | 4.16 | 1.33 | 2.28 | 0.02 |
|  | female | 97 | 34.27 | 4.00 |  |  |  |
| Upper lip length  (mm) | male | 100 | 18.84 | 1.99 | 1.00 | 3.69 | <0.01 |
|  | female | 97 | 17.84 | 1.80 |  |  |  |
| Lower lip length  (mm) | male | 100 | 15.08 | 2.31 | 0.49 | 1.62 | 0.11 |
|  | female | 97 | 14.59 | 1.93 |  |  |  |
| Height of chin  (mm) | male | 100 | 24.62 | 3.01 | 1.42 | 3.41 | <0.01 |
|  | female | 97 | 23.20 | 2.84 |  |  |  |
| Facial depth  (mm) | male | 100 | 88.18 | 8.45 | 2.97^#^ | 2.52 | 0.01 |
|  | female | 97 | 85.21 | 8.06 |  |  |  |
| Nasal depth  (mm) | male | 100 | 20.25 | 3.98 | 1.21 | 2.25 | 0.03 |
|  | female | 97 | 19.04 | 3.58 |  |  |  |
| Prominence of nose  (mm) | male | 100 | 8.43 | 1.85 | -0.13 | -0.49 | 0.63 |
|  | female | 97 | 8.56 | 2.04 |  |  |  |
| Prominence of upper lip  (mm) | male | 100 | 3.61 | 1.09 | 0.52 | 3.22 | <0.01 |
|  | female | 97 | 3.08 | 1.18 |  |  |  |
| Prominence of lower lip  (mm) | male | 100 | 3.61 | 1.24 | 0.56 | 2.97 | <0.01 |
|  | female | 97 | 3.05 | 1.41 |  |  |  |
| Prominence of chin  (mm) | male | 100 | 3.14 | 1.91 | 0.19 | 0.74 | 0.46 |
|  | female | 97 | 2.95 | 1.57 |  |  |  |

n: sample size, #: clinical significance (mean difference greater than 2° or 2 mm)

**Supplementary document 3a: Correlations between the angular and linear facial traits in the mixed dentition stage**

**
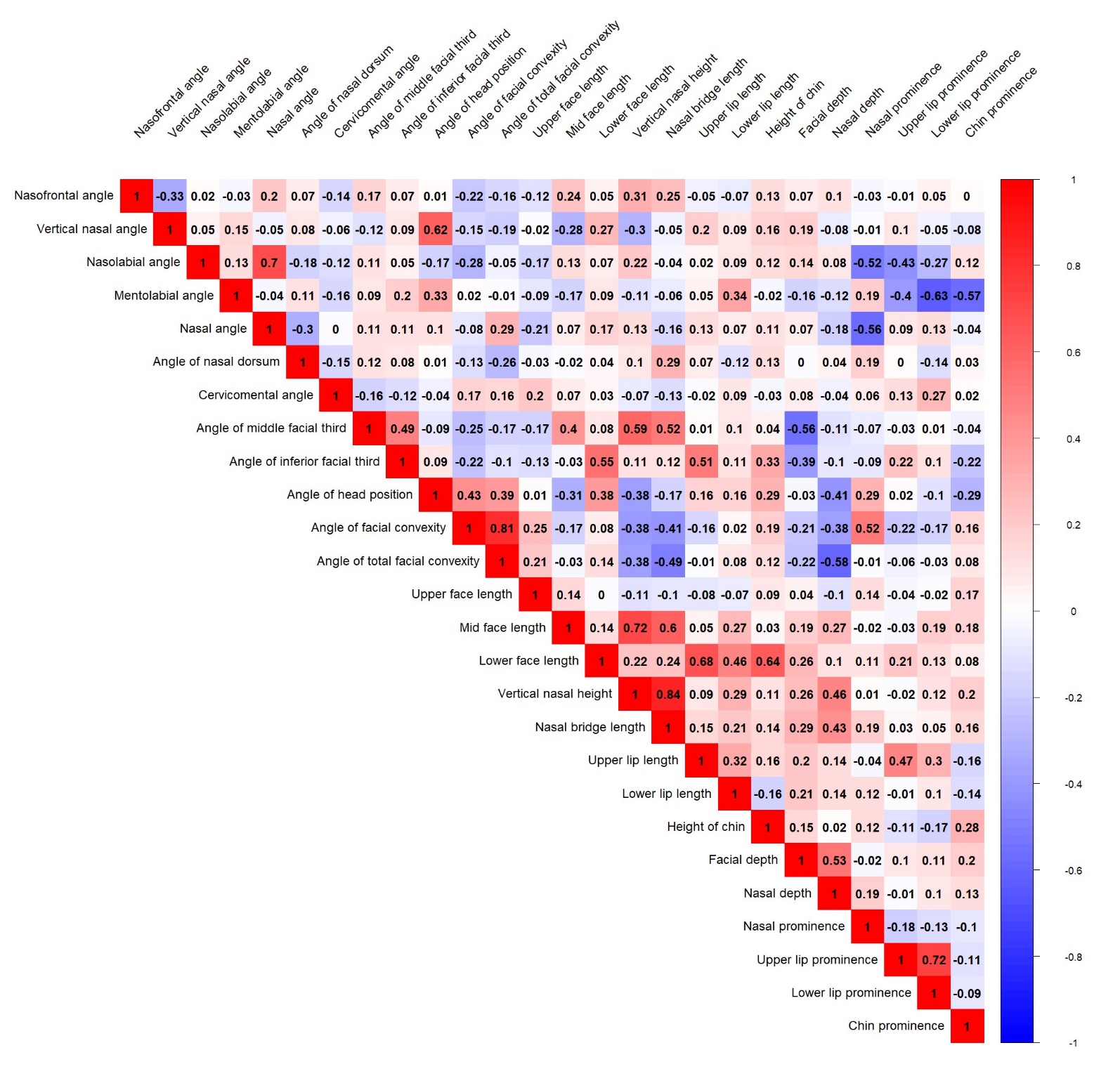
**

**Supplementary document 3b: Correlations between the angular and linear facial traits in the permanent dentition stage**


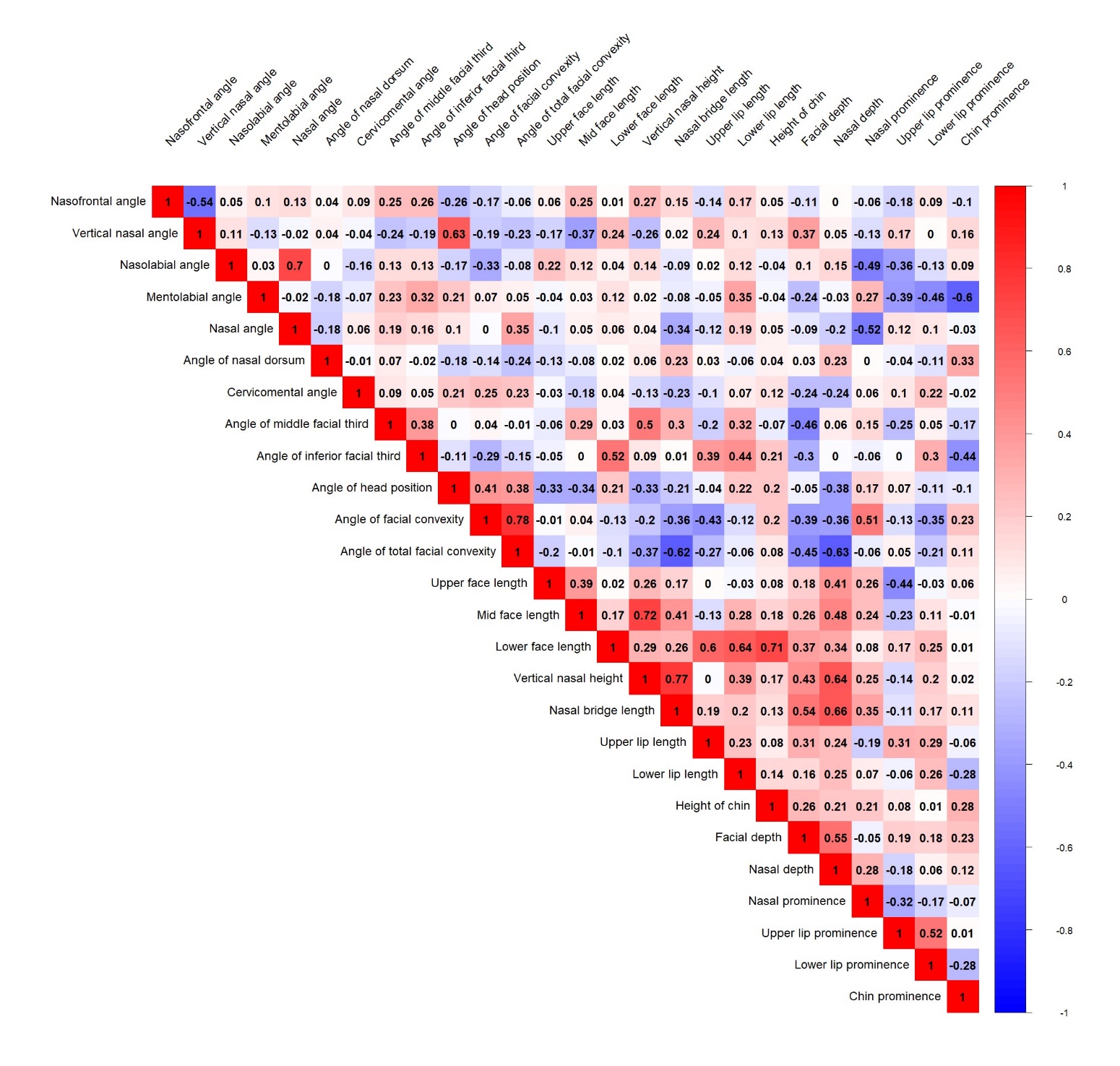

Supplement: cjae045_suppl_Supplementary_Material [file cjae045_suppl_supplementary_material.docx]
